# Supplementary material for: A Simple Elimination of the Thermal Convection Effect in NMR Diffusiometry Experiments
Source: Molecules. 2022 Sep 27;27(19):6399. doi: 10.3390/molecules27196399 (PMC9573581; doi:10.3390/molecules27196399)
Supplement: Supplementary file 1 [file molecules-27-06399-s001.zip › molecules-1924049-supplementary.pdf]

Supplementary Material

Figure S1: Representative data and the fitting parameters

Since we obtained 4 apparent diffusion constants from the four peaks of the NaDS the averaged value was used for further analysis. We had 24 diffusion experiments for Figure 2 of the manuscript therefore almost one hundred data were fitted with the MestreNova software. In order to illustrate the quality of the data we show below randomly selected spectra form each concentration

This are the comment of Mnova software about the fitting results:
NOTE: The Probnotmono value is important to assess whether or not the model for the fit we are assuming, in this case, a three parameter-exponential model satisfies. For instance, if you assume a mono-exponential model but the experimental data follows a bi or multi-exponential behavior, then the analysis will not be fully correct.On the other hand, if we assume that the model is mono-exponential, the 'rError' value gives the [absolute] probable error in the value of 'r', where 'r' is:
y = a*exp(-**r***x)+b

We applied the above equation in the following form
B+F*exp(-x*G) therefore the rError is for *G*, the apparent diffusion coefficient.
We always checked the B/F*100 values (offset %). It was not more than 1% in either cases.

Diffusion of NaDS 0.015 mol kg^{-1} solution in D_2O Δ=30 ms, δ =8 ms

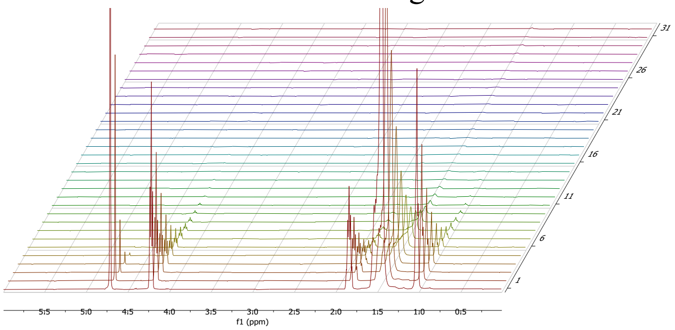

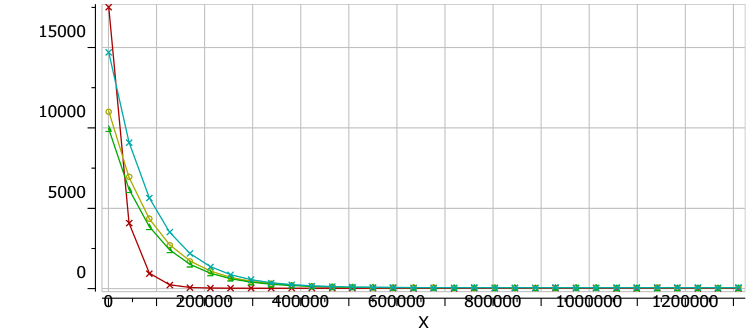

Water peak (4.71 ppm)
B= 13.5723, F= 17857.2, G= 3.46422e-05
rError = 2.81893e-08, probnotmono = 0.983053
CH_2 4.22 ppm
B= 34.7125, F= 11074.3, G= 1.11381e-05
rError = 1.89289e-08, probnotmono = 0.92135
CH_2 1.84 ppm
B= 39.9349, F= 9954.4, G= 1.13319e-05
rError = 1.68905e-08, probnotmono = 0.96145
CH_3 1.03 ppm
B= -77.8792, F= 15200.9, G= 9.92067e-06
rError = 1.06481e-07, probnotmono = 0.993336

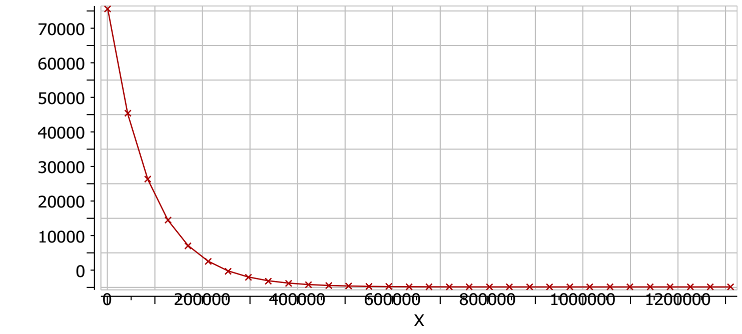

CH_2 peak (1.45-1.55 ppm)
B= -3475.41, F= 490054, G= 1.05753e-05
rError = 4.97833e-08, probnotmono = 1

Diffusion of NaDs 0.005 mol kg^{-1} solution in D_2O Δ=15 ms, δ =8 ms

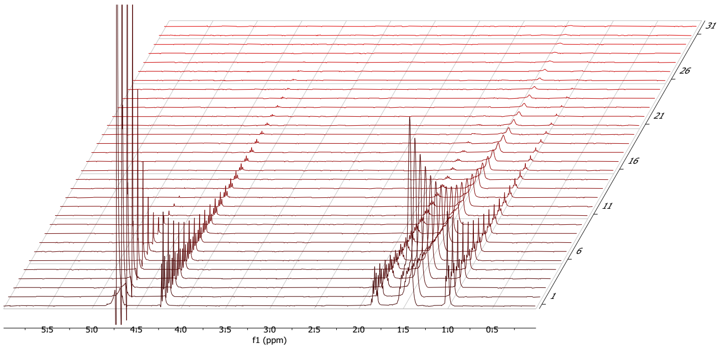

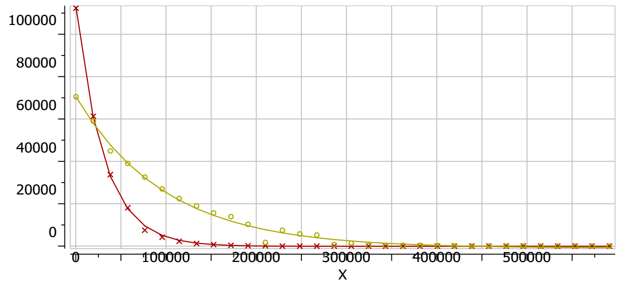

Water peak (4.71 ppm)
B= -104.773, F= 113720, G= 3.32785e-05
rError = 1.7288e-07, probnotmono = 0.999998

CH_2 1.42-1.53 ppm
B= -927.287, F= 71540.7, G= 1.00499e-05
rError = 2.32229e-07, probnotmono = 0.999796

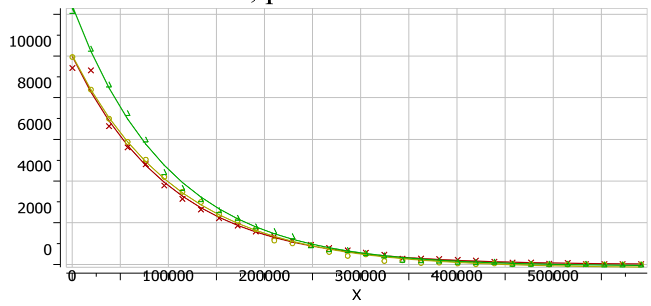

CH_2 4.22 ppm
B= -6.47894, F= 10001.7, G= 9.7169e-06
rError = 2.34496e-07, probnotmono = 0.92135

CH_2 1.84 ppm
B= -172.298, F= 10207.8, G= 9.06488e-06
rError = 9.56075e-08, probnotmono = 0.999269

CH_3 1.03 ppm
B= -77.8792, F= 15200.9, G= 9.92067e-06
rError = 1.06481e-07, probnotmono = 0.993336

Diffusion of NaDs 0.074 mol kg[-1] solution in D_2O Δ=30 ms, δ =8 ms

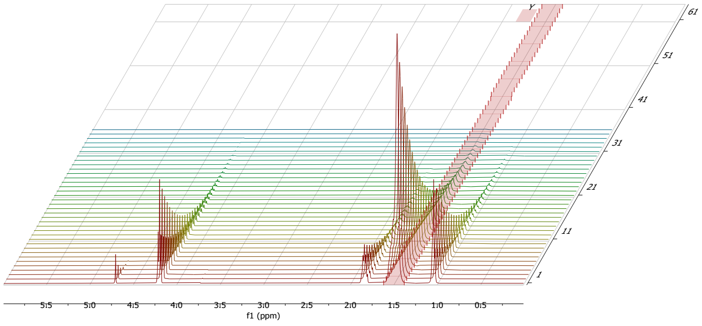

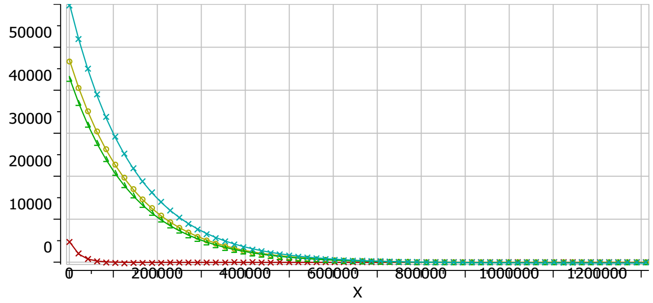

Water peak 4.71 ppm

B= -36.605, F= 4903.88, G= 3.36471e-05

rError = 8.87396e-07, probnotmono = 1

CH_2 4.22 ppm

B= -124.228, F= 47283, G= 7.01776e-06

rError = 1.30441e-08, probnotmono = 1

CH_2 1.84 ppm

B= -121.582, F= 43192.5, G= 7.0181e-06

rError = 1.23635e-08, probnotmono = 1

CH_3 1.03 ppm

B= -163.557, F= 60490.6, G= 6.96134e-06

rError = 1.22043e-08, probnotmono = 1

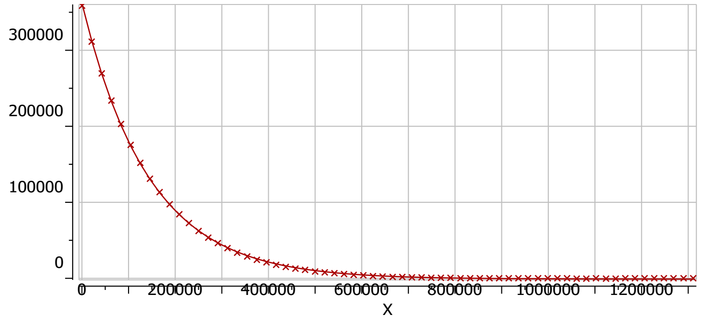

CH_2 peak 1.43-1.53 ppm

B= -999.561, F= 362961, G= 6.96679e-06

rError = 1.22093e-08, probnotmono = 1

Diffusion of NaDs 0.047 mol kg^{-1} solution in D_2O Δ=20 ms, δ =8 ms

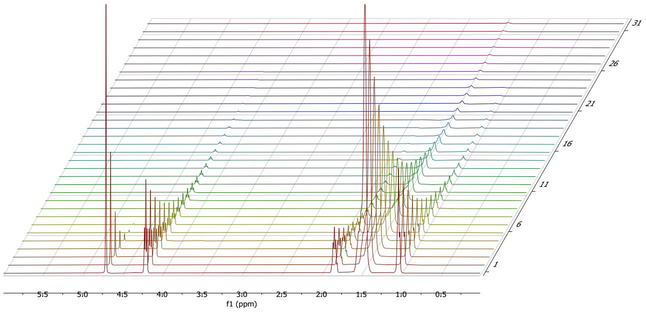

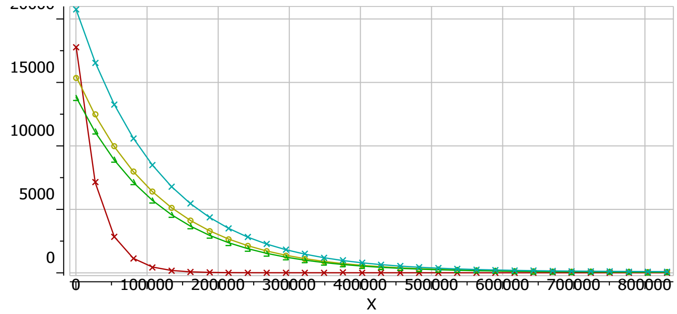

Water peak 4.71 ppm

B= 11.6092, F= 17995.2, G= 3.4249e-05

rError = 3.98459e-08, probnotmono = 0.855578

CH_2 4.22 ppm

B= 45.0069, F= 15466.4, G= 8.2649e-06

rError = 1.68678e-08, probnotmono = 0.997661

CH_2 1.84 ppm

B= 53.8717, F= 13825.4, G= 8.29809e-06

rError = 1.00387e-08, probnotmono = 0.92135

CH_3 1.03 ppm

B= 94.9812, F= 20698.5, G= 8.38078e-06

rError = 5.72934e-09, probnotmono = 0.855578

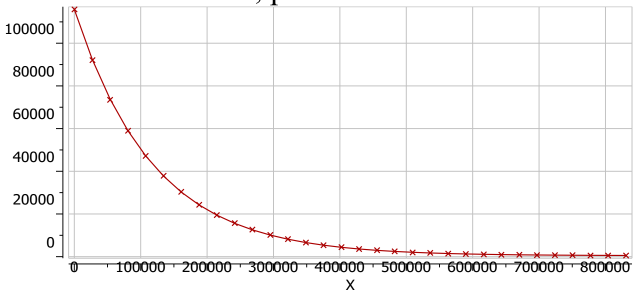

CH_2 peak1.43-1.53 ppm

B= 455.465, F= 115408, G= 8.40559e-06

rError = 8.11708e-09, probnotmono = 0.99995

Diffusion of NaDs 0.059 mol kg^{-1} solution in D_2O Δ=30 ms, δ =8 ms

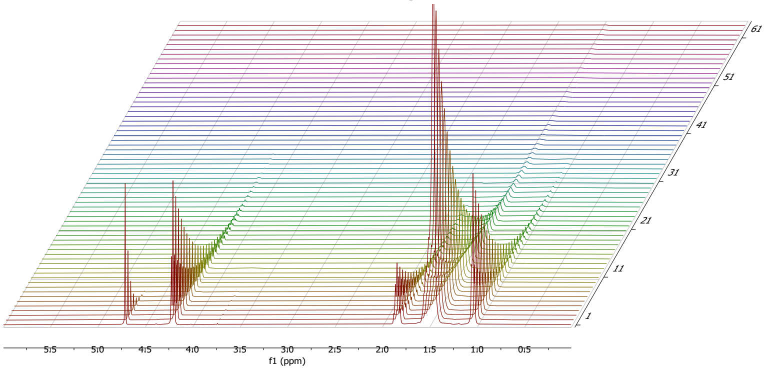

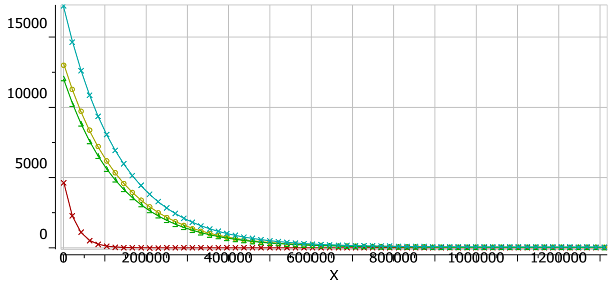

Water peak 4.71 ppm
B= 0.388892, F= 4737.32, G= 3.47174e-05
rError = 5.7734e-08, probnotmono = 0.97725

CH_2 4.22 ppm
B= 22.1, F= 13105, G= 7.24064e-06
rError = 7.68164e-09, probnotmono = 1

CH_2 1.84 ppm
B= 28.7228, F= 11992.2, G= 7.32302e-06
rError = 6.8353e-09, probnotmono = 0.99702

CH_3 1.03 ppm
B= 48.8479, F= 17119.7, G= 7.28536e-06
rError = 6.72099e-09, probnotmono = 0.987776

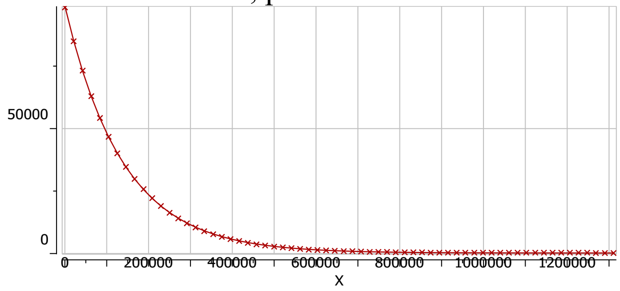

CH_2 peak1.43-1.53 ppm
B= 213.327, F= 98876.2, G= 7.2341e-06
rError = 4.61981e-09, probnotmono = 1

Diffusion of NaDs 0.029 mol kg^{-1} solution in D_2O Δ=30 ms, δ =8 ms

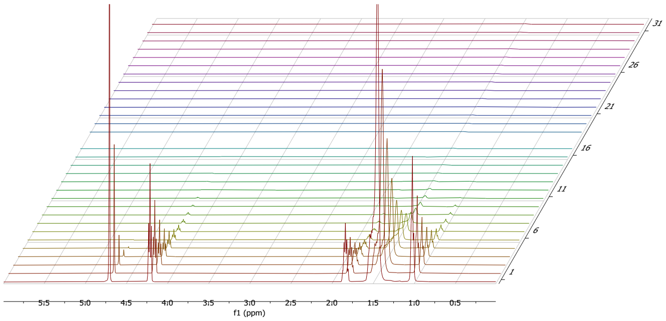

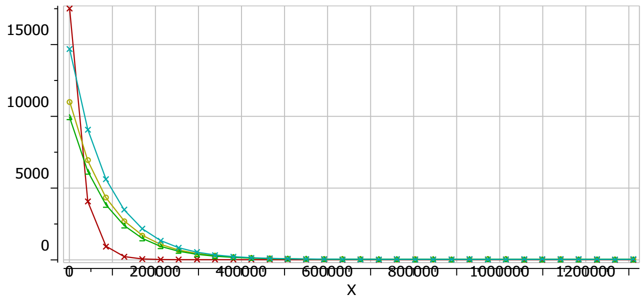

Water peak 4.71 ppm
B= 13.5723, F= 17857.2, G= 3.46422e-05
rError = 2.81893e-08, probnotmono = 0.983053

CH_2 4.22 ppm
B= 34.7125, F= 11074.3, G= 1.11381e-05
rError = 1.89289e-08, probnotmono = 0.92135

CH_2 1.84 ppm
B= 39.9349, F= 9954.4, G= 1.13319e-05
rError = 1.68905e-08, probnotmono = 0.96145

CH_3 1.03 ppm
B= 34.2499, F= 14751.2, G= 1.14127e-05
rError = 9.54809e-09, probnotmono = 0.92135

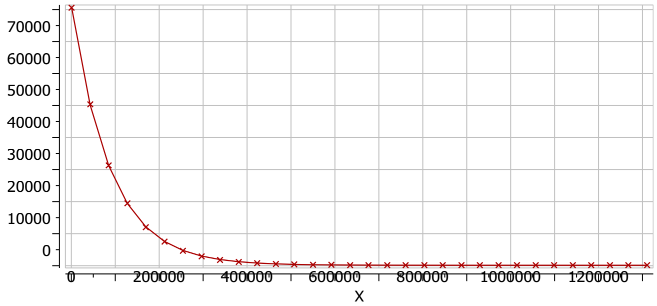

CH_2 peak1.43-1.53 ppm
B= 136.632, F= 81115.2, G= 1.12706e-05
rError = 1.23238e-08, probnotmono = 0.999998

Illustration of the determination of real diffusion coefficients.

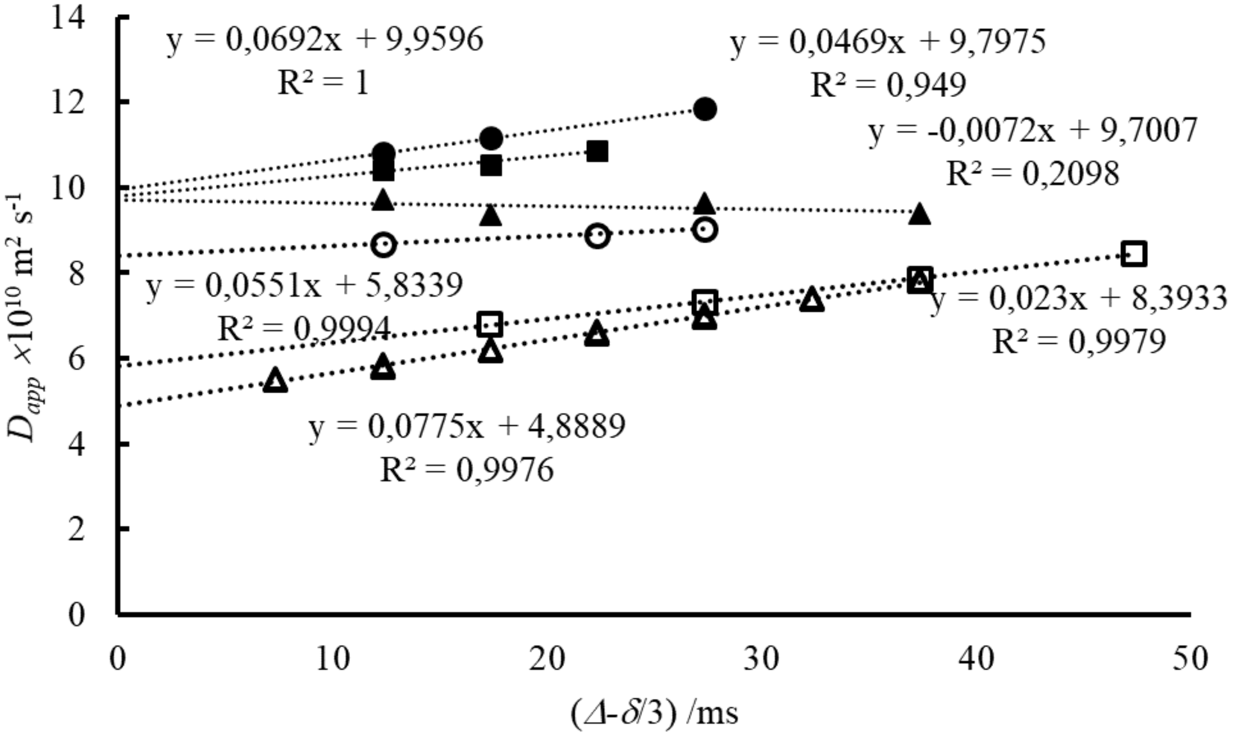

Figure S2. The apparent self-diffusion coefficients measured at different concentrations as a function of the diffusion time (Δ) at 319 K. Concentrations: ▲ 0,005 mol kg^{-1}, ● 0.015 mol kg^{-1},. ■ 0.029 mol kg^{-1}, ○ 0.047 mol kg^{-1}, □ 0.059 mol kg^{-1}, Δ 0.074 mol kg^{-1}

The extrapolation of apparent diffusion coefficients to zero diffusion time. The values are written in the Figure in that order as the lines are drawn except the value of 0.047 mol kg^{-1} which is at the most right position.
